# Combined Management for Maxillary Lateral Incisor With Open Apex and Type 2 Palatogingival Groove—A Case Report (4‐Year Follow‐Up)

**DOI:** 10.1155/crid/9503936

**Published:** 2026-07-28

**Authors:** Swapnil Chandekar, Shishir Singh, Gaurav Kulkarni, Padmini Sekhar

**Affiliations:** ^1^ Department of Conservative Dentistry and Endodontics, Terna Dental College, Navi Mumbai, Maharashtra, India, ternadental.org

**Keywords:** maxillary lateral incisor, palatogingival groove, radicular groove, root canal system, root variation

## Abstract

This case details the clinical management of a maxillary right lateral incisor with a Type 2 palatogingival groove extending beyond the middle third, in association with the root canal system. Despite a poor prognosis, the tooth was successfully managed through a combined nonsurgical and surgical endodontic approach; the underlying clinical protocol is outlined in this case report, with the 4‐year clinical follow‐up showing postoperative osseous healing.

## 1. Introduction

The palatogingival groove (PGG), or radicular groove, is a developmental anomaly on the lingual surface of maxillary incisors, first described by Lee et al. in 1968 [1–3]. It originates near the palatal aspect of anterior teeth and often extends towards periapex facilitating bacterial entry to the pulp and periapical tissues [4, 5]. Its incidence ranges from 2.8% to 18%. Thought to result from enamel organ or HERS infoldings, it is sometimes considered a mild form of dens invaginatus [6]. A simple PGG involves only partial root structure, whereas a complex PGG extends to the apex, often communicating with the pulp or forming an accessory root. Early detection and cervical management are crucial to avoid periodontal and pulpal issues. This case report discusses a multidisciplinary approach of treating a Type 2 PGG in Tooth #12 [1].

## 2. Case Report

A 27‐year‐old female reported to the Department of Conservative Dentistry and Endodontics with pain in the maxillary anterior region and gave a history of childhood trauma in the same area 15 years ago due to a domestic fall (Figure 1a,b).

**Figure 1:**
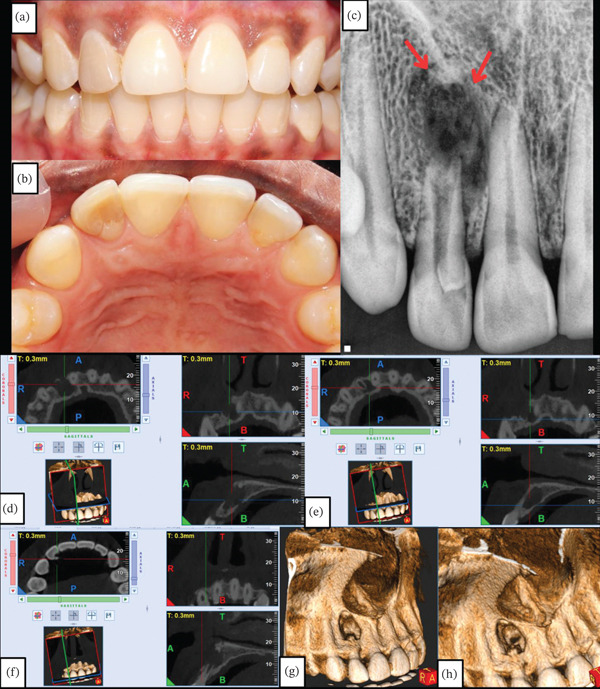
(a, b) Preoperative intraoral photograph of maxillary right lateral incisor showing labial and palatal view with PGG. (c) Preoperative periapical radiograph (red arrows) showing extensive periapical radiolucency. (d–h) CBCT analysis of maxillary right lateral incisor.

### 2.1. Clinical Examination

Tooth #12 (maxillary right lateral incisor) presented with a grayish‐white labial discoloration and sensitivity to percussion, with no clinical signs of caries, sinus tract, or crown fracture noted. The patient reported a history of childhood trauma in the region. Pulp testing (Kerr Electric Pulp Tester, United States) revealed no response in Tooth #12, with normal responses in adjacent teeth. Palpation was nontender. Periodontal probing showed normal depths except for a localized 5‐mm palatal pocket associated with a Type 2 PGG shallow groove that extends beyond the coronal third with a complex system [1] (Figure 2). Periapical radiograph and CBCT revealed apical root resorption and a well‐defined periradicular radiolucency (Figure 1c–h). The patient was informed in detail about the proposed treatment plan and its course, and written informed consent was obtained. The patient was also informed that the case would be reported for academic purposes, and written consent for publication was obtained.

**Figure 2:**
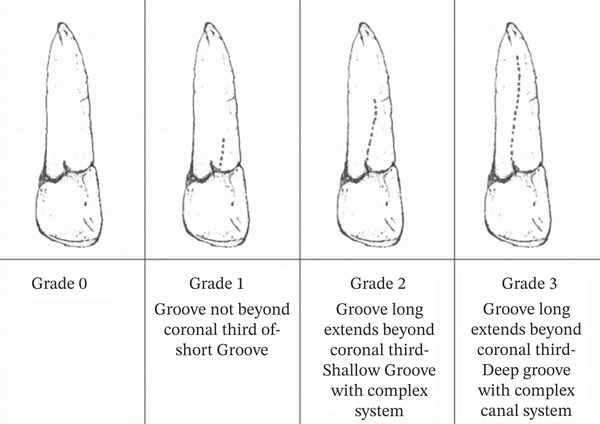
Classification of radicular groove/palatogingival groove (Gu et al. [1]).

#### 2.1.1. Phase 1

Primary root canal therapy was initiated with access gained and working length determined using an electronic apex locator (Colten CanalPro Apex Locator, Coltène/Whaledent, Switzerland) and confirmed by digital radiography (Nanopix‐1 Rvg, Orikam, Eighteeth, Changzhou, China). The canal was cleaned and shaped using the crown‐down technique up to apical size #120 K File (Mani, INC. Japan). Irrigation was done with 5% sodium hypochlorite and 17% EDTA (Prime Dental Products, Mumbai, India). Calcium hydroxide (RC‐Cal, Prime Dental Products, Mumbai, India) was used as interappointment intracanal medicament for 7 days, and access cavity was sealed using eugenol free temporary restoration (Cavit‐G, 3M ESPE, United States). Obturation was completed with a custom‐made master roll cone (Meta BioMed, Korea) using cold lateral compaction, followed by postendodontic restoration with resin composite, LUNA Nano Hybrid (Southern Dental Industries [SDI], Bayswater, Australia) (Figure 3a–d).

**Figure 3:**
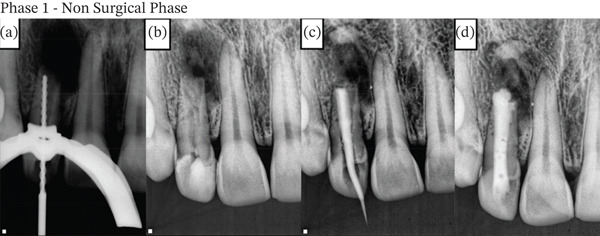
Phase 1: root canal treatment. (a) Working length estimation, (b) intracanal medicament (calcium hydroxide), (c) master cone, and (d) obturation.

#### 2.1.2. Phase 2

The second phase involved root‐end surgery and saucerization of the PGG, which was taken into consideration after 3 days of postobturation and performed under strict aseptic conditions. Local anesthesia with adrenaline of 2 mL each (1:80,000 Lignox 2% INDOCO, India) was administered via infraorbital and nasopalatine nerve blocks. The overlying soft tissue including mucosa and periosteum was reflected as a full‐thickness flap and a bony window was created using a straight micromotor handpiece (NSK, Japan) and size‐04 tungsten carbide round bur (SS White, Lakewood, United States) under copious saline irrigation (Figure 4a–c). The defect was curetted, and a tissue sample was collected for histopathological analysis. A 3‐mm retrograde cavity was prepared using ultrasonic microsurgical tips (Figure 4d,e) (Woodpecker, China) and confirmed via intraoral periapical radiograph (Figure 4f) (Nanopix‐1 Rvg, Orikam, Eighteeth, Changzhou, China). Mineral trioxide aggregate (MTA; Angelus Repair HP, Brazil), mixed per manufacturer instructions, was placed with an MTA carrier and condensed using retrograde pluggers into the retrograde cavity (Figure 4g,h) and confirmed using radiograph (Figure 4i). For the PGG, a palatal flap was raised and reflected. A microsurgical mirror aided in visualizing the defect, which was then saucerized using (NSK‐S max) surgical airrotar handpiece and fissurotomy diamond bur (SS White, Lakewood, United States) and sealed with light‐cured restorative glass ionomer cement (GC Type‐9 GIC, Japan). (Figure 4j–l) Bleeding was controlled using (Botroclot Topical Solution, Juggat Pharma, India), and followed by (Buccal, Palatal) flaps closure with the help of simple interrupted 5‐0 Mersilk sutures (Ethicon, Johnson & Johnson MedTech, India) along with immediate postoperative x‐ray to check the uniformity of retrograde filling postsuture placement (Figure 4m–o). Postoperative instructions were given, and the patient was scheduled for suture removal after 1 week. As a definitive restorative measure to mask the tooth discoloration, all‐ceramic (lithium disilicate) crown was placed after 3 weeks of Phase‐2 to ensure long‐term tooth stability.

**Figure 4:**
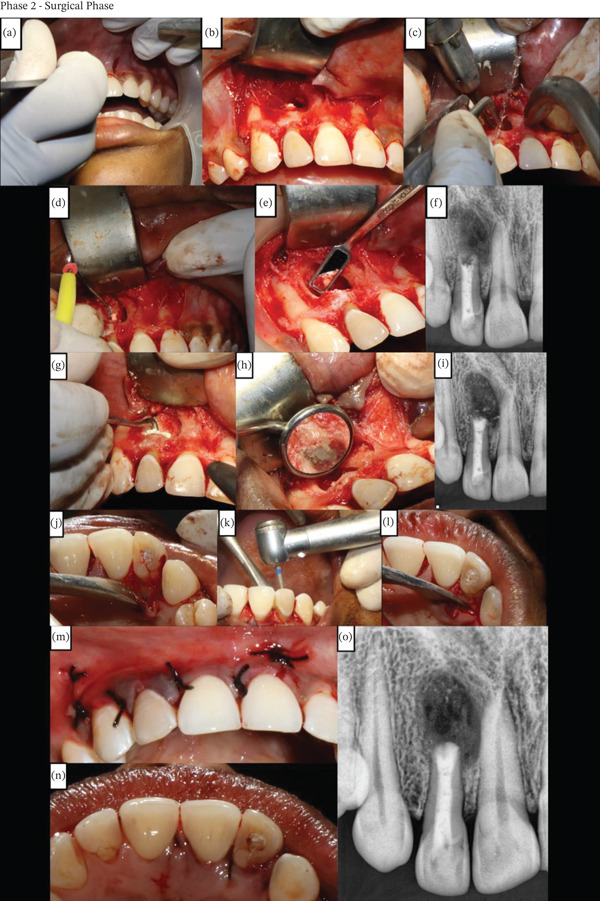
Phase 2: root end surgery and PGG management. (a) Incision. (b) Reflection of flap. (c) Lesion curettage. (d, e) Retrograde cavity preparation. (f) X‐ray of retrograde cavity. (g, h) Placement of root end filling material MTA. (i) Postoperative x‐ray of MTA placement. (j–l) Management of palatogingival grove—reflection of palatal flap, saucerization of PGG, and restoration of PGG using GIC. (m, n) Postoperative placement of interrupted sutures labial and palatal aspect. (o) Immediate postoperative x‐ray following retrograde filling MTA and saucerization of PGG with GIC.

## 3. Discussion

The selected treatment plan is aimed at creating an apical seal using a biocompatible material in the open apex, eradicating bacterial plaque through saucerization of the PGG, sealing the coronal portion of the groove to prevent recolonization, and promoting periodontal regeneration and clinical improvement (Figure 2). The PGG, acting as a “plaque trap,” can facilitate endodontic‐periodontal lesions and mimic conditions like vertical root fractures on radiographs, complicating diagnosis. Prognosis depends on groove severity and periodontal damage; mild cases may respond to odontoplasty and periodontal therapy, while severe cases require more complex intervention [7–9]. In this case, custom‐fit gutta‐percha was used to obturate the open apex, as the tooth was presented with open apex, and there was no apical stop for termination of root filling. Custom‐made (tailor made) gutta‐percha was constructed by combining number of heated, coarse, gutta‐percha butt to tip on a clean and sterilized glass slab [10], which was followed by root‐end surgery and retrograde filling with a 5‐mm MTA plug, chosen for its bioactivity and sealing ability [10]. Studies have shown that a 5‐mm thick apical MTA barrier has proven to be significantly stronger with lesser leakage than a 2‐mm thick barrier. MTA releases calcium and hydroxyl ions in phosphate‐containing fluids, forming hydroxyapatite, and offers superior sealing compared with gutta‐percha, without compromising root strength [11]. However, due to the potential for MTA displacement, GIC was used to seal the groove, given its chemical bonding ability and promotion of tissue attachment [12–14], which was followed by full coverage crown prosthesis (Emax Lithium Disilicate) to mask the grayish‐white discoloration of crown. The patient was recalled for follow‐up after 4 years, The 4‐year recall interval was influenced by both clinical stability and patient factors. The patient remained symptom‐free and did not report any issues that would mandate an earlier review. Consequently, the clinical opportunity to reassess the case only arose after 4 years, at which point both radiographs and CBCT confirmed sustained periapical healing. Clinical examination showed satisfactory healing. At the 4‐year recall, the patient exhibited a stable periodontal condition. Clinical evaluation revealed healthy gingival tissues, normal probing depths, no bleeding on probing, and no evidence of periodontal pocketing or attachment loss. The surrounding periodontium appeared to be well‐maintained, indicating favorable periodontal stability throughout the follow‐up period (Figure 5a,b), and comparative radiographs (Figure 6a–c) demonstrated evident reduction in the periapical lesion with periapical scar (Figure 6c). CBCT analysis further confirmed progressive resolution of the periapical radiolucency, indicating favorable periapical healing (Figure 6). Healing following the interdisciplinary endodontic‐periodontal treatment highlights the value of a comprehensive approach in managing complex cases involving developmental groove.

**Figure 5:**
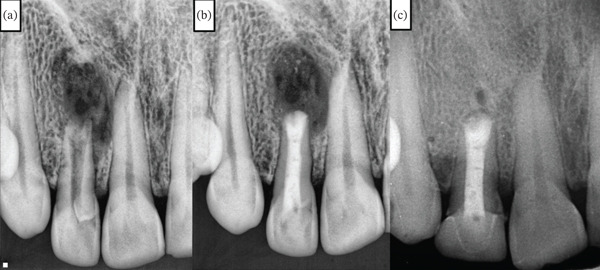
Comparative x‐rays. (a) Preoperative x‐ray. (b) Immediate postoperative x‐ray. (c) Four‐year follow‐up x‐ray.

**Figure 6:**
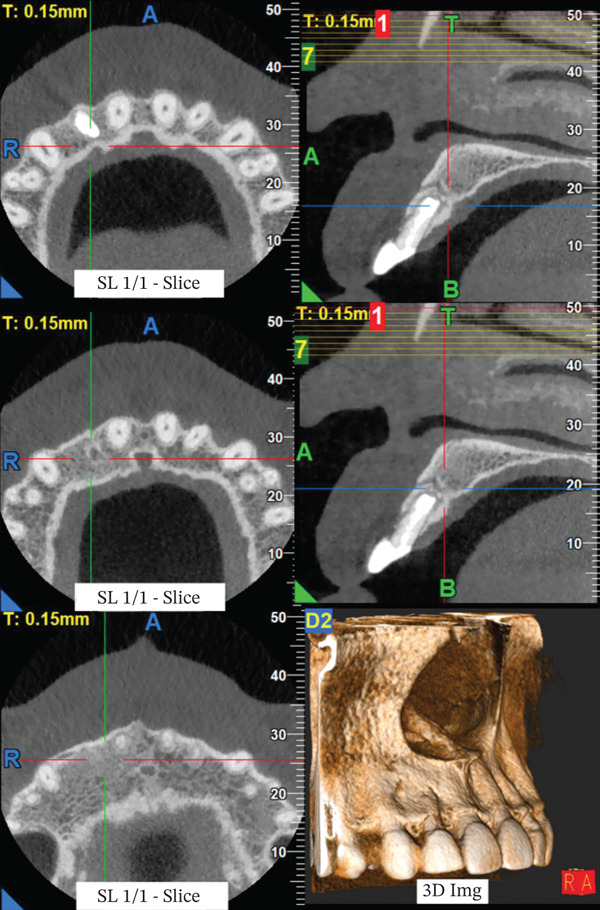
Four‐year follow‐up CBCT analysis with evidence of osseous healing and periapical scar.

## 4. Conclusion

This case highlights the successful management of a Type 2 PGG through a combined endodontic and surgical approach. Early diagnosis, effective debridement, sealing of the groove, and use of biocompatible materials like MTA and GIC were key to achieving favorable clinical and radiographic outcomes. A multidisciplinary strategy is essential for preserving teeth affected by such developmental anomalies.

## Funding

No funding was received for this manuscript.

## Conflicts of Interest

The authors declare no conflicts of interest.

## Data Availability

The data that support the findings of this study are available on request from the corresponding author. The data are not publicly available due to privacy or ethical restrictions.
